# National Getis-Ord G_i_*statistics datasets for select populations, 2019-2023 American community survey 5-year estimates

**DOI:** 10.1016/j.dib.2026.112550

**Published:** 2026-02-06

**Authors:** Cyanna McGowan, Shaina J. Alexandria, Kiarri N. Kershaw

**Affiliations:** Department of Preventive Medicine, Feinberg School of Medicine, Northwestern University, 680 North Lake Shore Drive, Suite 1400, Chicago, IL 60611, USA

**Keywords:** Residential segregation, Neighborhoods, Census tract, Spatial data

## Abstract

Racial or ethnic residential segregation is the physical separation of individuals into different neighborhoods by race or ethnicity. This selective sorting process has a large impact on the social, economic, political, and health outcomes of populations, with more marginalized populations having worse outcomes. Segregation measures seek to quantify this process and characterize how different population groups are spread across geographical areas. Residential segregation has traditionally been measured at the metropolitan or county level, but a growing number of population health studies have quantified it at the neighborhood or “local” level. Many such studies have used racial or ethnic composition as a proxy for local residential segregation. However, racial or ethnic composition does not take into account the larger context surrounding the neighborhood individuals live in The G_i_*statistic is the most commonly used spatial local segregation measure in the literature. However, it is hard and time consuming to compute, potentially discouraging researchers from its use. Thus, we provide G_i_*statistics for the entire US for 2019–2023 American Community Survey 5-year estimates, for numerous population groups. We also provide R code that can be used to calculate the G_i_*statistic for years before 2023.

Specifications Table

**Table:** 

Subject	Social Sciences
Specific subject area	Spatial Data, Residential Segregation
Type of data	*Table (.csv format)*
Data collection	Census bureau data were collected from the IPUMS National Historical Geographic Information System (NHGIS). The 2019–2023 5-year American Community Survey estimates were collected.
Data source location	*Chicago, Illinois*
Data accessibility	Repository name: National Getis-Ord Gi* statistics for select populations; 2019–2023 Data identification number: **openicpsr-235,349** Direct URL to data: https://www.openicpsr.org/openicpsr/project/235349/version/V1/view
Related research article	none

## 1 Value of the Data

• Unlike commonly used metrics such as racial/ethnic composition, the G_i_* statistic measures neighborhood level segregation relative to the composition of the larger metropolitan area. This refinement better represents the granularity of variation in racial residential segregation and acknowledges that the impact of segregation may depend on the composition of surrounding neighborhoods.
• Spatial measures like the G_i_* statistic offer advantages over aspatial measures because they account for uncertainty around the boundaries that are used to operationalize neighborhoods, such as census tracts. Despite its theoretical appeal, the use of spatial measures such as the G_i_* statistic can be hard and time-consuming to compute, requiring additional software and pre-processing of data. This creates a barrier on the use of spatial measures in the literature, as investigators must choose between easily computed aspatial measures, and its more robust spatial counterpart. Providing researchers with these measures across multiple racial and ethnic groups and years of data removes this key barrier to operationalizing segregation as a spatial construct.
• These datasets and accompanying R code produce G_i_*statistics for the entire country allowing for researchers to compare across time and geographic location. Because the census tract is a commonly used administrative boundary in the US, researchers will be able to link these data to a wide variety of other data sources, such as electronic health records, cohort studies, nationally representative cross-sectional studies, and other Census/American Community Survey data.

## 2 Background

Racial and ethnic residential segregation is the systematic exclusion of marginalized groups from certain neighborhoods and concomitant resources and opportunities [1]. The link between greater residential segregation and poorer health outcomes have been well documented in the literature [[2], [3], [4], [5], [6]]. Residential segregation has traditionally been measured at the metropolitan or county level, but a growing number of population health studies have quantified it at the neighborhood or “local” level. Currently, racial or ethnic composition (e.g., percent Black) is the most commonly used local residential segregation measure [7]. However, this measure does not consider the composition of the larger area that the neighborhood is situated in It is also an aspatial measure, meaning it does not incorporate information from surrounding local contexts beyond a given administrative boundary (e.g., census tract) that individuals might also regularly encounter or consider to be part of their neighborhood. The local Getis-Ord G_i_* statistic addresses these limitations by measuring the extent to which a focal neighborhood and its surrounding neighborhoods, differ from a larger areal unit [7,8]. However, while this measure does use publicly available census data, it can be difficult to calculate. Thus, we have calculated and publicly shared G_i_*statistics for all census tracts and numerous population groups.

## 3 Data Description

The G_i_* statistic returns a Z score for each neighborhood (census tract), indicating the extent to which the racial/ethnic composition in the focal tract and neighboring tracts deviates from the mean racial composition of a larger areal unit. Higher positive G_i_* Z scores indicate higher racial/ethnic segregation or clustering (overrepresentation), scores near 0 indicate racial integration, and lower negative scores suggest lower racial/ethnic representation (underrepresentation), in comparison with the racial composition of the larger areal unit.

This article describes the G_i_* statistic datasets in the linked repository. The repository includes two tables in .csv format and one .docx word document. Each .csv file utilizes data from the 2019–2023 American Community Survey 5-year estimates and provides versions for two methods of measuring contiguity (Queen and Rook).

Each dataset is organized in the same way and contains the same nine columns. Each row represents a census tract based on the 2020 U.S. Census definition. Each census tract is identified by its Federal Information Processing Standard (FIPS) code. A FIPS code is a unique numeric identifier issued by the National Institute of Standards and Technology (NIST) to ensure uniform identification across all geographic units. Each FIPS code is comprised of 11 digits, representing a census tract that is identified by a two-digit state code, three-digit county code, and a six-digit tract code. Each file includes the G_i_*statistic for each census tract as well as the number of neighboring tracts that were used for the calculation, determined by either Queen or Rook contiguity weights. Rook contiguity defines neighbors by those that share a common edge only, while Queen contiguity neighbors are those that share both an edge or a ``corner'' (common vertex) [9]. Additionally, either the Core-Based Statistical Area (CBSA) or County was used as the larger areal unit. Areal units were determined by geographic boundaries defined by the census bureau. A CBSA refers collectively to metropolitan and micropolitan statistical areas. It is made up of the county or counties belonging to at least one urban core of at least 10,000 population, and the adjacent communities that have a high degree of integration with the urban core [10]. The vast majority of census tracts fall within a CBSA, however a small percent of census tracts do not fall within this administrative unit, and thus the County the tract belongs to was selected as the larger areal unit instead. All focal tracts were row-standardized and “self-included” and counted as a neighbor (see Experimental Design section for more details). It should be noted that the estimation of the queen contiguity is consistent across software packages (e.g., R and ArcGIS), but there is variation in how much a focal tract must be touching a neighboring tract to be counted as a “common edge” for the rook contiguity [11]. For more information, see the Spdep and Creating Neighbors R package descriptions. Each file contains four racial/ethnic groups. Racial categories were defined by the 1997 Office of Management and Budget (OMB) standards on race and ethnicity and were self-reported at the time of the survey [12].

Lastly, the repository includes one .docx file which describes the data. An overview of file names, file type, file description, and column descriptions are given in Tables 1 and 2. If the user is interested in additional data, we provide G_i_*statistics for prior years in a separate repository (openicpsr-170,541).

**Table 1:** Title and description of files in ICPSR deposit main workspace.

File	Name	Type	Folder	Description
1	Gstat23_RookContiguity.csv	.csv table	Main Directory	2019–2023 5-year ACS values, rook contiguity
2	Gstat23_QueenContiguity.csv	.csv table	Main Directory	2019–2023 5-year ACS values, queen contiguity
3	Documentation on calculating the Local G_i_* statistic	.docx	Main Directory	Documentation on calculating the G_i_* statistic

**Table 2:** Column names and descriptions of .csv tables in main workspace.

Variable name	Description
FIPS_ID	Census tract identifier (does not include leading zero)
nhWhite_Gstat	G statistic for non-Hispanic White Adults
nhWhite_n_neigh	Number of neighboring tracts considered for nhWhite_Gstat
nhBlack_Gstat	G statistic for non-Hispanic Black Adults
nhBlack_n_neigh	Number of neighboring tracts considered for nhBlack_Gstat
nhAsian_Gstat	G statistic for non-Hispanic Asian Adults
nhAsian_n_neigh	Number of neighboring tracts considered for nhAsian_Gstat
Hispanic_Gstat	G statistic for Hispanic Adults
Hispanic_n_neigh	Number of neighboring tracts considered for Hispanic_Gstat

**Fig. 1:**
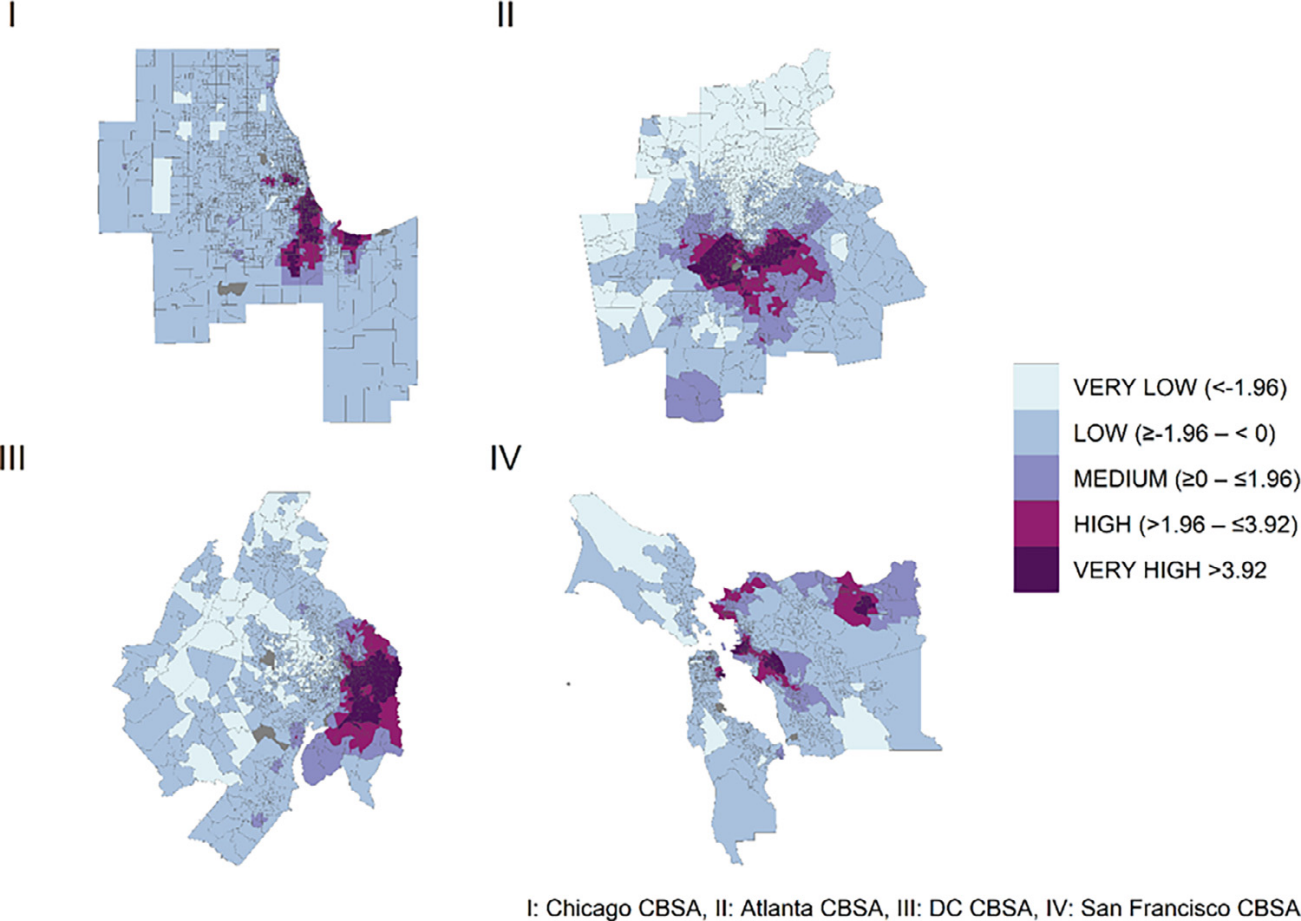
Distribution of the G_i_* statistic across four CBSA’s among Non-Hispanic Black Adults.

To incorporate the G_i_* statistic into data analysis users may spatially join the G_i_* statistic values to census tract polygon data. Users should first obtain a tract level shapefile in 2020 values. Shapefiles are easily accessible through the US Census Bureau. Users may then join data from the .csv table to the 2020 shapefile. This enables spatial analyses and visualization of segregation among different areas (e.g., Fig. 1). By using the G_i_* statistic in this way, researchers can gain valuable insight into the spatial trends and distribution of segregation.

Fig. 1 visually illustrates the geographical distribution of segregation across different CBSA’s.

## 4 Experimental Design, Materials and Methods

Calculating Getis-Ord G_i_* statistic involves numerous computational steps, including:

1. Sourcing data from the American Community Survey,
2. Selecting population groups and creating proportion variables,
3. Adding and aligning tract and CBSA geography,
4. Determining larger areal unit,
5. Creating spatial weights matrix objects,
6. Looping through each larger areal unit present in the dataset,
7. Assigning indicator values for census tracts that could not compute a G_i_* statistic.

A “*gstat*” package was created for R (version 4.2.1) to combine all computational steps needed to compute the statistic. This package expands on the *localg* function from the spdep package in R. The *localg* function takes on numerous parameters that must be created separately before using the function, such as weight matrices and row-standardization. Additionally, the function will only take on one larger areal unit at a time. The *gstat* package was created to combine all steps into one function call, reducing errors and creating a more time efficient process.

All variables needed for the *gstat* function were sourced from ACS table B03002: Total Population, using the *get_acs* function from the *tidycensus* package. After loading the *gstat* package, a vector of variables is created for 11 populations of interest that may be used in the package parameters. Variable names are based on how they are labeled in the ACS table. For example, non-Hispanic Black is represented by the variable name B03002_004. Additionally, a vector of values representing all 50 states and DC, as well as five other helper functions are loaded.

The *gstat* function has four parameters: *geoType, endYear, raceGroups*, and *queen*. The *geoType* parameter takes on a character string that represents the geography level at which the G_i_* statistics will be calculated. To produce the data available in the repository, “tract” was chosen in order to output G_i_* statistics at the census tract level. This also represents the “neighborhood” or smaller focal unit. To determine the larger focal unit, the *gstat* package will determine which tracts will use a CBSA, and which tracts will use the County they are situated in The package makes this determination after census tracts have been spatially joined to their CBSA boundaries. If a tract spatially falls within a CBSA, they will be assigned a CBSA code, if a tract does not fall in a CBSA boundary, their CBSA code will be left blank. This lets the package know that these tracts will need to use the county FIPS code that is assigned to all tracts. Once it is time to compute the G_i_* statistics, CBSA and county tracts were split and run through the same program separately and then bound back together at the end. The next parameter is *endYear* and takes on an integer value that represents the last year of 5-year ACS estimates. To compute G_i_* statistics based on data from the 2019–2023 5-year ACS estimates, 2023 was selected for this parameter. The *endYear* also determines the year that the geographic units will be standardized to. The 2019–2023 ACS estimates are standardized to 2020 units, meaning both census tract and CBSA assignments are based on their 2020 definitions. Next, the *raceGroups* parameter determines the population(s) of interest. This parameter accepts a character string or group of strings representing one or more of 11 racial/ethnic groups loaded earlier. For the data available in this repository, non-Hispanic White, non-Hispanic Black, non-Hispanic Asian, and Hispanic populations were chosen for the *raceGroups* parameter. The *queen* parameter represents either queen or rook contiguity. To apply the contiguity, a spatial weights matrix is created that assigns a weight of 1 to all neighboring tracts, and a weight of 0 to all other tracts, quantifying the spatial relationship between the focal tract and their neighbors. These weights are additionally row-standardized by dividing each assigned neighbor weight by the sum of the total weights assigned to each neighbor. This is used to create proportional weights since not all census tracts have an equal number of neighbors. Additionally, all tracts are self-included meaning they are counted as their own neighbor and given a value of 1 in the matrix. For this repository, the function was run once with queen = TRUE and once with queen = FALSE, producing both queen and rook contiguity estimates. Lastly, a small number of census tracts are not able to compute a G_i_* statistic and are considered missing for any analysis. For information purposes, the “missing” G_i_* statistics are represented in the data by indicator codes that describe why the value is missing and is summarized in the table below Table 3.

**Table 3:** Description and Indicator codes for missing Gi* statistics.

Reason for missing value	Indicator code
Only one tract in the CBSA/County	−99
Zero population in the focal tract	−100
No target population in the CBSA/County	−199
Tract is connected to every other tract in the CBSA	−299
All tracts are islands (no connections)	−399

## Limitations

These data include limitations related to data availability. This repository only includes data for one 5-year period and does not include all racial/ethnic groups available for use. Additionally, these data are only available in 2020 geographic boundaries and thus limit longitudinal associations with earlier years of data. However, the R code used to produce these data can be accessed through the NU Social Environment and Health lab GitHub (https://github.com/NU-SocEnvLab/local-gstat), allowing researchers to access a more comprehensive set of data.

## Ethics Statement

Our study does not involve human subjects, animal experiments, or any data collected from social media platforms. Therefore, we confirm that we have read and strictly adhere to the guidelines for authors provided by Data in Brief in terms of ethical considerations.

## CRediT authorship contribution statement

**Cyanna McGowan:** Software, Validation, Writing – original draft, Writing – review & editing, Visualization. **Shaina J. Alexandria:** Software, Writing – review & editing. **Kiarri N. Kershaw:** Conceptualization, Validation, Writing – review & editing.

## Data Availability

ICPSRGstat Queen and Rook contiguity, 2019-2023 estimates (Original data).
